# Puerperal Eclampsia

**Published:** 1882-05-20

**Authors:** L. S. Manning

**Affiliations:** Manchester, Ky.


### PUERPERAL ECLAMPSIA.

BY L. S. MANNING, M. D., MANCHESTER, KY.

By way of preface I should say that the physician in this country is his own druggist and carries his supplies with him. What he does not have in the way of drugs or instruments he does without, since he can neither buy nor borrow for emergencies. Hence. the doctor, when he starts on a twenty-mile ride, has to select what he shall take and what he shall leave. To the patient it is far more important that he should take quinine than that he should take a thermometer; that he should take veratrum viride, rather than a sphygmograph. So when your country practitioner has made a selection of drugs, and crammed into his bags a few plasters, with a very few of the most common instruments, he has a load for his "one horse." These circumstances account for the omission of some important details in the histories following; and moreover, when at this terrible juncture the friends of the patient, in great anguish, are appealing to you for succor, if you do not lose your presence of mind, your attention will be wholly occupied with efforts of relief, rather than in the application of instruments of precision.

CASE I.—March 4th, 1874. Mrs. D., white, married, æt. 20, primapara. Saw her at 5 a. m. Has sore mouth and headache. Skin moist, pulse normal. Bowels and bladder just evacuated. Recurring pains at intervals of five minutes. Digital examination reveals the os uteri thin, dilating, dilatable, vertex presenting but not yet engaged in superior strait. 9 a. m. Very little advancement. Pains more frequent and stronger. Without warning, had a convulsion at 11.20 a. m., which lasted three minutes. After this administered Squibb's strong ether, by inhalation, but at 12 m. she had another convulsion. Ruptured membranes, used forcible dilatation, and pushed the ether, but not to the extent of complete anæsthesia. 2 p. m. Labor advancing; withheld ether, when she went into another convulsion. After this kept her under the partial influence of ether till the birth of a living male child, weighing nine pounds, at 3.30 p. m. Had used f. ℥xij ether. Everything was arranged for the mother's comfort, but at 4 p. m. had another convulsion. Gave—

℞ Chloral .................................... gr. xv,
Potass. bromid ................................ gr. xx.

In syrup, once in three hours. At 4.30 p. m. she lay moaning, skin hot, pulse 120. At 6 p. m. another convulsion.

March 5th. No more convulsions. Patient could recollect none of the occurrences of the previous day. Mother and child doing well. Uninterrupted recovery.

CASE II.—April 5th, 1875. At 9 a. m. called to Mrs. W., married, multipara, æt. 24. Complains somewhat of frontal headache. Pains have been getting stronger and more frequent for some hours. Examination revealed os uteri dilating and dilatable. Had entered on second stage of labor, vertex presenting, first position. Three or four pains delivered a living female child with cord around its neck. Severed cord and passed the child to a nurse. In twenty minutes removed secundines entire. I should have stated that during third stage of labor gave fl. ext. ergot, ʒj, with brom. pot., grs. xxx. Applied binder, and everything was arranged for the mother's comfort. We were sitting around congratulating her upon her happy delivery, when, without any warning, she had one of those dreaded convulsions. When she began to breathe naturally again, administered chloroform by inhalation, to partial anæsthesia, whenever patient manifested restlessness, till 1 p. m., March 6th, when I gave her pulv. Doveri, grs. viij, which was vomited. At 4 a. m. the same was repeated and was retained. At 7 a. m., gave brom. pot., grs. xxx, with tr. verat. virid., gtt. vj, and repeated the same in four hours. 6 p. m. Has had several hours' quiet sleep. Recollects none of the occurrences of the previous night after the convulsion.

April 7th. Mother and child doing well.

April 8th. Secretion of milk established. From this time on recovery was rapid and complete.

CASE III.—May 7th, 1878, S. B., colored, unmarried, primipara, aged 19, very robust build; would weigh, perhaps, 185 pounds; attendants say she began staggering about and talking like a crazy person, twelve hours previous to my visit, 9 a. m. Had had a number of fits; none could tell how many. Was now comatose, tongue swollen and protruding, breathing laborious, frequent, stertorous; pulse 90 and small. Tied up the arms and opened a vein in each, at the elbow. Let the blood, which was of a very dark color, flow until it ceased spontaneously, when about ℥xxx had been withdrawn. Found the pulse had improved Attempted forcible dilatation of os uteri, but without success.

℞ Chloral,.................................... gr. x,
Pot. brom.,.................................. gr. xx,

In syrup every three hours. At 5 p. m. no more convulsions; breathing improved; tongue withdrawn; some feeble labor-pains, and labor had advanced to second stage; vertex presenting; fœtus evidently dead. If I had owned obstetric forceps, I would have delivered my patient at this time, but was forced to leave all to nature, only giving a cathartic to stimulate expulsive pains.

May 7th. During the past night she was delivered of a dead fœtus; still unconscious, and has evacuated the bladder and rectum in bed, without warning. Ordered her to have ergot and quinine.

May 9th. Consciousness has returned, but recalls nothing of the events of the past three days. Complains of frontal headache, with great stiffness and soreness all over.

She made a rapid recovery.

CASE IV.—February 19th, 1880. F. W., single, white, primipara, robust and healthy. Had been complaining of headache and feeling badly for a day or two. Was taken with convulsions at 4 a. m., and I saw her at 2 p. m.; just passed through a convulsion. Tongue swollen and protruding, bleeding in places; breathing labored, frequent and stertorous; examination showed first stage of labor begun, os uteri thin, dilating and dilatable. Tied up both arms and punctured veins at elbow. The right arm bled to ℥xx, the left refused to bleed:

℞ Brom. pot.,................................ gr. xxx.
Morph. sulph.,............................ gr. ½.

Every hour. Gave chloroform by inhalation, and attempted to turn and get hold of the feet, but without success. Chloroform was pushed whenever she manifested great restlessness. At 4:30 p. m. second stage of labor had been completed, and having no forceps with which to deliver the child. I made an incision in the vertex of the already dead fœtus, and inserting my finger, soon accomplished delivery.

I now found hour-glass contraction of the womb, but with a little time and considerable exertion, delivered secundines entire; at the same time stimulating the uterus to contract by kneading. At 5:30 p. m. tongue withdrawn; breathing much improved; pulse soft and compressible.

℞ Calomel, .................................. gr. ij,
Sulph. morph., ............................ gr. ¼.

Every six hours.

February 20th. Consciousness returned during the night. She has no recollection of the occurrences of the previous day. Symptoms favorable.

February 25th. Recovered.

CASE V.—November 28th, 1880. Called to see Mrs. M., æt. 28, white, married, the mother of four children, the youngest seven days old. In her confinement had been attended by a midwife, and nothing unusual was noticed, except the patient was despondent, and frequently spoke of dying. She also had persistent headache; had no dropsy, nor had received any injury. Forty-eight hours before I saw her all thought she was doing remarkably well, when suddenly she gave a loud scream, followed by another. She said her head had burst, that she was going to die, and implored her husband to take good care of the children. Shortly after this she had a terrible convulsion, requiring the strength of three men to hold her in bed, biting her tongue, and frothing at the mouth. She never regained consciousness, and had convulsions, recurring at intervals of less than an hour, for the next eighteen hours. When seen by me she had had no convulsions for five or six hours; skin cold and clammy; breathing rapid and superficial; pulse almost imperceptible; eyes fixed and staring. In a few minutes death ended the scene. No *post-mortem.*

CASE VI.—June 1st, 1881. Mrs. W., white, married, fourth pregnancy. Saw her at 7 a. m. Attendants said she had had two hard fits, about half an hour apart; that it still lacked two or three weeks of her term; that she had passed large quantities of urine the night before; that she had complained very much lately of persistent frontal headache; that just before each fit she had vomited bile mixed with mucus and saliva. She now vomited again, and attendants warned me she would have a fit. In a few moments I saw her eyes become fixed, her face drawn strongly over the left shoulder. Then commenced one of those terrible convulsions seen only in puerperal eclampsia, with her face drawn strongly to the left. Convulsion lasted between three and four minutes, patient biting her tongue and frothing at the mouth; remaining cometose several minutes after convulsion ceased.

As this patient was not in robust health, I resolved to try the morphia treatment. I, therefore, dissolved sulph. morph. grs. ij, and gave at once, *per orem.* Upon examination I found feet and legs slightly œdematous, os uteri high up, thin, dilated to the size of half a dollar. Made strenuous efforts at further dilatation, with some success. Head presentation—head not large, and pelvis roomy. At 9 a. m. she began vomiting again, and, fearing another convulsion, I applied chloroform to her nose; but it was too late. She went off into another fearful convulsion, her head this time drawn strongly to the right. I now tied up her arm and bled to ℥xx. I also gave hypodermic injection of one and one-half grains sulph. morph. into the cellular tissue, over deltoid muscle. This made her drowsy, and I made still further efforts at dilatation. At 11 a. m., her respirations numbered ten per minute, pupils contracted, pulse 80 and full. She made another effort at vomiting, followed by convulsion. I now had the temerity to dissolve two grains more of sulph. morph. and administer *per orem*. At 1 p. m. patient profoundly comatose, respirations eight per minute. Without premonition she had a violent convulsion. After this had ceased the outlook was gloomy, indeed. Respirations sighing, as if each one would be the last, and numbered only four per minute. By my attempts at dilatation the respirations improved slightly, and at the end of an hour's hard work succeeded in applying long forceps and delivering a living female child weighing seven pounds. Child seemed fully developed, was cyanotic, and respiration was established with some difficulty. The placenta being removed, the mother was put to bed. Respirations four per minute, and sighing. Kept up artificial respiration by elevating arms above the head and bringing them back again to the sides for some hours. Gave strong, black coffee, and stimulating doses of carb. ammon. with quinine.

June 2d, 8 a. m. Patient has just recovered consciousness; recollects nothing since first convulsion; respirations almost normal; pulse 60, full volume; complains of headache, and of being sore and stiff all over. The child lived but twelve hours.

June 3d. Patient continued to complain of her head for some weeks, and, aside from an occasional cathartic, she had,

℞ Potass. bromid.,.............................. gr. xx,

Tr. hyoscyam.,............................ f. ʒss,

Tr. nuc. vom.,............................. gtt. v.

In syrup, three times a day.

June 30th. Patient able to be about the house, though still feeble and complaining of her head. At this writing she enjoys her usual health.

I offer a few deductions of my own, the value of which each one may determine for himself:

1st. The division of puerperal convulsions into three classes, as is done by some obstetricians, is entirely without foundation. Hysterical convulsions are hysteria; epileptic convulsions are epilepsy; apoplectic convulsions are apoplexy; while puerperal convulsions, or eclampsia, are *sui generis*, and without relief, tend to death.

2d. I consider Case 5 to be the course of pureperal eclampsia when unchecked by medical art. I have the first case to hear from that recovered without aid.

3d. In Case 6, I would have the reader note; (*a*) The head drawn first to one side and then the other, showing that the irritant is equally in both hemispheres of the brain; proving as I believe, that these attacks are not apoplectic; (*b*) That no amount of morphia compatible with life will arrest all cases of puerperal eclampsia.

4th. Note the frontal headache is a prodro ne in every case; and in every case where the fact was noted at all the os uteri was thin and stubborn in character, and the labor pains were irritating and inefficient.

5th. The temperature was not taken in these cases, but I do not think there was great departure from the normal in a single case, unless it was lowered.

6th. If the urine had been tested after convulsions were established, doubtless albumen would have been found, as it is in all cases in which respiration is interfered with, and hæmatesis is imperfect.

7th. I would treat pureperal women with persistent headache by the exhibition of brom. pot. and nux vom. At inception of labor would give xx grain doses of chloral. After a convulsion I would bleed, deliver as rapidly as possible, give chloroform by inhalation at evidences of recurring convulsions, since they recur rhythmically, and the time of one seizure passed over, there is a lull, so to speak, until the next period of excitation. I would use chloroform so long as convulsions threatened, and use only moderate doses of opiates or other drugs.—*Med. and Surg. Reporter.*

